# Cannabis and Cognitive Functioning: From Acute to Residual Effects, From Randomized Controlled Trials to Prospective Designs

**DOI:** 10.3389/fpsyt.2021.596601

**Published:** 2021-06-10

**Authors:** Josiane Bourque, Stéphane Potvin

**Affiliations:** ^1^Department of Psychiatry, Perelman School of Medicine, University of Pennsylvania, Philadelphia, PA, United States; ^2^Department of Psychiatry and Addiction, Faculty of Medicine, University of Montreal, Montreal, QC, Canada; ^3^Centre de Recherche de l'Institut Universitaire en Santé Mentale de Montréal, Montréal, QC, Canada

**Keywords:** cannabis, delta-9-tetrahydrocannabinol, cognition, longitudinal design, memory

## Abstract

In recent years, several jurisdictions have revised their regulation policy toward both medical and recreational use of cannabis. These changes have elicited concerns regarding how legalization impacts academic achievement and work performance. This review evaluates the acute and long-term (residual) association between cannabis use and cognitive functioning that underlies poor academic and work performance. Relative to other reviews, this article focuses on cross-over randomized controlled trials and prospective designs given that they allow to test the impairing effects of cannabis exposure at the within-subject level. Acute cannabis cognitive effects are discussed separately for known confounding factors such as levels of delta-9-tetrahydrocannabinol (Δ^9^-THC), Δ^9^-THC:cannabidiol ratio, previous cannabis use and, comorbidity with psychosis-spectrum disorders. The cognitive residual effects of cannabis are detailed in relation to duration of abstinence, frequency of use, comorbidity with psychosis-spectrum disorders, types of cognitive domains assessed, and age of cannabis use initiation. Moreover, considering the fact that adequate longitudinal studies can make inferences about causality between cannabis use and impaired cognitive functioning when disentangling between-subject from within-subject variation, proofs for the three main non-mutually exclusive hypotheses about this relationship will be presented: i) the cognitive vulnerability hypothesis as part of the more general common antecedent hypothesis, ii) the concurrent cannabis impairing hypothesis, and iii) the neurotoxic hypothesis of cannabis. Current research provides evidence for mild to moderate acute cannabis effects on episodic and working memory, processing speed, and executive functions. Mild residual impairing effects were also observed in these exact same cognitive domains, suggesting that adverse effects following cannabis intoxication persist at least days or weeks following cannabis abstinence. Relative to adult-onset, adolescent-onset cannabis use seems to explain the dose-response relationship and is associated with longer lasting residual effects even in mild users (<weekly). The association between cannabis and cognition is likely explained by common antecedents, such that genetic and shared environment factors predispose individuals to both cannabis use and cognitive deficits, and to a lesser degree, neurotoxic effects.

## Introduction

In recent years, several jurisdictions have revised their regulation policy toward both medical and recreational use of cannabis. These changes have elicited concerns regarding how state and federal legislations impact cannabis use prevalence. In addition to the Canadian legalization of recreational use in 2018, more than 30 US states have legalized medical cannabis use, and more than 10 states have legalized its recreational use. In adult populations (>26 years old), evidence points toward increases in frequency of use and in rates of cannabis use disorders (CUD) pre- to post-medical and recreational laws (1, 2). The literature evaluating adolescent cannabis users is more complex (1, 3, 4). Recreational, but not medical legalization, seems to positively affect cannabis use prevalence, and only the most severe form of cannabis misuse (i.e., CUD) is affected by legislation changes (1, 3, 5, 6).

Another concern is the marked increase in concentrations of delta-9-tetrahydrocannabinol (Δ^9^-THC), the principal psychoactive agent contained in cannabis, since the 1970s and most specifically since the last decade. Concentrations of Δ^9^-THC ranged between 0.5 and 4.0% in the 1970s, whereas contemporary strains from North America, Europe, and Australia attain concentrations of 15% and over (7–11).

A renewed interest in understanding the potential adverse effects of cannabis use from a public health perspective has emerged following these changes in regulatory policy and cannabis potency. One such potential adverse effect is its impact on cognitive functioning, which may translate into lower academic achievement (12–15), decreased work performance (16, 17), and a rise in the number of motor vehicle accidents (18–20). Increasingly, studies show that adolescence may be a particularly vulnerable period for the cognitive effects of cannabis use. The known psychoactive effects of cannabis are exerted through its two main components, Δ^9^-THC and cannabidiol (CBD), and their action on the endogenous cannabinoid system. The endocannabinoid system is also tightly involved in neurodevelopmental processes such as neuronal specification, migration and maturation, axonal elongation, and synaptogenesis; processes that continue to occur during adolescence (21). Consequently, it has been proposed that the effects cannabis exert on cognition would be more deleterious if age of onset occurred during adolescence.

It is therefore imperative to review the literature investigating the potential effects of cannabis use on cognitive functioning to inform the public, as well as stakeholders. The first part of this article offers a narrative review of studies examining the acute effects of cannabis. An emphasis is placed on understanding the contribution of specific confounding factors such as the content in Δ^9^-THC of cannabis products, the Δ^9^-THC:CBD ratio, previous cannabis use, and comorbidity with psychosis-spectrum disorders. Considering that acute effects are most robustly examined with double-blind cross-over randomized controlled trials (RCT) which mitigate potential sources of experimental bias by testing effects at the within-subject level, the section on acute effects primarily discusses findings from these cross-over experiments, unless specified otherwise. In a second section, we discuss the residual effects (or long-term effects following abstinence) of regular cannabis use with a focus on both meta-analyses of cross-sectional studies and longitudinal studies. This second section will review how (i) duration of abstinence, (ii) frequency of use, (iii) psychosis-spectrum comorbidity, (iv) types of cognitive domains assessed, and (v) age of cannabis use initiation interact with the residual cognitive effects of cannabis. Considering the fact that adequate longitudinal studies can make inferences about causality between cannabis use and impaired cognitive functioning when disentangling between-subject from within-subject variation, proofs for the three main non-mutually exclusive hypotheses about this relationship will be presented: (i) the cognitive vulnerability hypothesis as part of the more general common antecedent hypothesis, (ii) the concurrent cannabis impairing hypothesis, and (iii) the neurotoxic hypothesis of cannabis.

## Acute Effects

Acute effects refer to those relative to exposure–that is, cannabis-induced intoxication. The vast majority of studies on acute effects report impaired cognitive performance following cannabis/Δ^9^-THC exposure. A recent meta-analysis including more than 52 studies and 1,580 healthy individuals shows that verbal learning and memory (e.g., encoding, consolidation, retrieval), and working memory are the cognitive domain most impaired by acute cannabis-induced intoxication (22). Indeed, exposure to Δ^9^-THC or cannabis extract exerts moderate cognitive deficits (effect sizes: g = 0.69; g = 0.51; g = 0.51, respectively), in these three domains (22). These results echo prior well-documented evidence of acute impairments in these domains, notably in humans (23) as well as in rodents and non-human primates (24). Administration of cannabis also seems to elicit mild to moderate adverse effects on processing speed (g = 0.38) and executive functioning (g = 0.37) (22). Lastly, the latter meta-analysis explored the effects of acute cannabis exposure on attention and inhibitory (i.e., response inhibition and decision making) performance and reported only mild detrimental effects (g = 0.24; g = 0.28, respectively) (22). Regarding the speed of processing domain, we found that the harmful effects of cannabis/Δ^9^-THC were smaller in the oral administration studies relative to studies using other routes of administration, including smoked administration (effects are reported in Table 1).

**Table 1:** Acute effects of cannabis use on cognitive functions.

**Cognitive domain**	**N studies**	**Effect size (g) 95% CI**
Attention	30	**−0.24 (−0.11**, **−0.36)**
Verbal learning	14	**−0.69 (−0.49**, **−0.89)**
Verbal memory	12	**−0.51 (−0.37**, **−0.65)**
Working memory	23	**−0.51 (−0.37**, **−0.66)**
Executive function	13	**−0.37 (−0.25**, **−0.49)**
Processing speed	38	**−0.38 (−0.28**, **−0.49)**
Impulsivity	14	**−0.28 (−0.17**, **−0.39)**

*CI, Confidence Interval*.

*Effects presented in bold are significant*.

*This Table has been adapted from Zhornitsky et al. (22). Effect sizes are negative, which means that decreases in cognitive performances were observed in users relative to non-users. An effect size of ~−0.2 is considered as small; an effect size of ~−0.5 is considered as moderate; an effect size of −0.8 is considered as large*.

One sub-domain of cognitive functioning that has recently received much attention is social cognition, which refers to a set of processes involving social interactions. These processes include mainly emotion recognition and the interpretation of others' emotional states (e.g., theory of mind). Among the few studies that investigated the acute effects of cannabis use on performance during social cognition tasks, some have reported impairments in emotional recognition of ambiguous faces (25) or threatening emotions such as fear and anger (26, 27), while this was not the case for other studies (28, 29). It is probable, but not certain, that exposure to Δ^9^-THC induces deficits in emotional recognition. Additional studies are needed to assess the quality of the evidence. As such, research linking cannabis use to impairments in theory of mind is insufficient and does not allow for the interpretation of potential effects on this sub-domain of socio-cognitive functioning.

### Δ^9^-THC Content

Cross-over designs have demonstrated that the effects of cannabis in infrequent users on several cognitive functions occur in a dose-response fashion (refer to Supplementary Table 1 for a summary of studies). For instance, it was demonstrated that for smoking, intravenous and oral administration of Δ^9^-THC, the higher dosage (or higher serum concentration) induced significantly more detrimental effects on verbal learning and memory, reaction times, and response inhibition relative to lower doses (30–35). Hart et al. (36) also found a dose-response relationship when investigating reaction times on various cognitive tasks, but not on performance accuracy when task time limit was not a factor. In addition to the absence of a time limit, this negative finding on performance accuracy from Hart et al. (36) could be explained by the fact that participants were daily users. Indeed, daily cannabis users often exhibit tolerance to the acute effects of cannabis on cognition (see section Previous cannabis use) and this may hinder efforts to demonstrate a dose-response relationship of cannabis on cognition.

Two studies have specifically investigated the effect of increasing concentration of Δ^9^-THC on decision making tasks (33, 37). The first demonstrated that the proportion of trials showing impairment increased as a function of serum concentration of Δ^9^-THC (33). The second found that only the higher dose yielded impairments relative to placebo (37). The failure to observe an effect at both doses in the second study may be due to the participants being daily users with tolerance to the impairing effects of cannabis and to the use of a small dose lower than reported to have an effect in occasional users.

Specifically for attention and working memory domains, the literature reports mixed findings: while most studies observed that the severity of impairments are a function of Δ^9^-THC content or performance is solely affected by the higher dose (30–32, 34, 35, 38, 39), some found that these domains were unaffected by Δ^9^-THC (32, 34, 36). Reconciliation of these contradictory findings is challenging considering the heterogeneity in the tasks used. A detailed analysis of 15 published studies assessing the dose effects of Δ^9^-THC on digit-span performance, demonstrated that negative results may be due to short task length (and low number of trials, e.g., 3-min Digit Span task), which imparts lower sensitivity to detect an effect compared to longer task durations (39). Altogether, there is converging evidence that the cannabis impairing effects on verbal learning and memory, response inhibition, and psychomotor speed occur in a dose-response fashion. The linear relationship between exposure to higher Δ^9^-THC content and worse performance on decision making, attention, and working memory were less robust, and are therefore probable at best.

### Δ^9^-THC:CBD Ratio

While Δ^9^-THC is responsible for the widely known psychoactive effects of cannabis (e.g., euphoria, psychological well-being, sensory experiences and appetite) (40), the effects of CBD are less well-understood. CBD is believed to be responsible for the anxiolytic and anti-inflammatory effects associated with cannabis use (41). When administered alone, without other cannabinoids, CBD may also have antipsychotic effects (41). What complicates research and generalizability of findings is that concentrations of Δ^9^-THC and CBD vary as a function of cannabis strains. For example, low doses of CBD can potentiate intoxicating Δ^9^-THC effects, while higher doses of CBD may reduce the intoxicating properties of Δ^9^-THC (42). As such, because of their different and sometimes even antagonistic properties (40), it is highly probable that Δ^9^-THC and CBD also exert distinct effects on cognitive functioning. To disentangle the ramification of these chemical compounds, an increasing number of experimental studies have specifically investigated the effect of different Δ^9^-THC:CBD ratios on cognition [(43), refer to Supplementary Table 2 for a summary of studies].

When investigating memory function (the cognitive domain most consistently impaired by cannabis), Schoedel et al. (44) observed that working memory performance (i.e., reaction times) was impaired by a high dose of synthetic Δ^9^-THC (dronabinol) compared to a placebo. However, performance following three different dosages of nabiximol (a compound with a Δ^9^-THC:CBD ratio of 1) was not different from placebo. On the contrary, in another within-subject cross-over design, administration of both Δ^9^-THC alone and Δ^9^-THC in combination with CBD induced deficits on episodic and working memory tasks. Only in the condition of exclusive CBD administration did subjects perform as well as during the placebo condition (45). The discrepancy in findings between these two studies could be explained by different Δ^9^-THC:CBD ratios, such that only at specific ratios does CBD attenuates the impairing effects of Δ^9^-THC. Between-subject designs provide further evidence of CBD attenuating the acute memory effects of Δ^9^-THC (46–48). For example, an experimental study exploring between-subjects contrasts found that healthy participants treated with placebo prior to receiving Δ^9^-THC presented poorer delayed but not immediate recall relative to baseline, while the group pre-treated with CBD showed no impairment (48). However, pre-treatment with CBD did not attenuate the deficits observed in other cognitive domains, such as working memory, psychomotor functioning and executive functions. Using a naturalistic design, studies have also reported that while individuals who used cannabis strains with lower CBD content had marked impairment on various memory tasks, those smoking cannabis high in CBD concentrations showed no performance deficits relative to the placebo condition, independent of Δ^9^-THC levels and baseline performance (46, 47).

Among other cognitive domains, Hindocha et al. (25) demonstrated that Δ^9^-THC exposure led to impaired emotional recognition when compared to both placebo and combined Δ^9^-THC and CBD conditions. For psychomotor function and driving performances, mixed evidence was found regarding the attenuating effect of CBD on Δ^9^-THC (45, 49, 50). Lastly, in an effort-related decision making task, CBD did not mitigate the impairing effect of Δ^9^-THC relative to placebo (51).

Altogether, CBD seems to dampen the deleterious cognitive effects of acute Δ^9^-THC exposure, for memory at the very least. While encouraging, these findings do not provide information on the potential long-term protective effects of higher CBD concentrations on chronic cannabis use. Unfortunately, this question remains difficult to address, even following legalization of cannabis use. Investigators would need to gather information on Δ^9^-THC and CBD concentrations in cannabis strains, in large cohorts of participants, followed longitudinally.

### Previous Cannabis Use

Another confound observed in the literature relating to the acute effects of cannabis is the users' status (e.g., non-/occasional users or regular/heavy users) (refer to Supplementary Table 3 for a summary of studies). Tolerance to the undesirable physiological effects of cannabis use among regular users was evidenced by RCT. Indeed, following Δ^9^-THC exposure, frequent users presented blunted perceptual alterations, psychotomimetic effects, anxiety, and increases in cortisol relative to occasional cannabis users, findings that could not be explained by group differences in plasma Δ^9^-THC (52). Five studies using a between-subject approach (difference between groups) of a cross-over placebo-controlled design have further investigated the presence of tolerance effects for the impairing effects of cannabis on cognition. Individuals with a cannabis use disorder (CUD), relative to non-users (i.e., <once/month), showed smaller Δ^9^-THC-induced impairments in immediate and delayed verbal memory tasks, while performing worse during the placebo condition (52). Similarly, administration of Δ^9^-THC (following pre-treatment with haloperidol) produced significant performance deficits on verbal learning and spatial working memory (not on verbal memory) in non-users specifically (53). However, Colizzi et al. (54) demonstrated that occasional and non-users did not perform differently on verbal memory during the drug condition. Of note, in this latter study, the authors failed to observe general Δ^9^-THC induced memory deficits across the whole sample. This negative finding could be explained by a lower sample size (*n* = 24 vs. 28 and 52) and/or the use of an intermediate oral dosage of Δ^9^-THC (10 mg; a dosage typically lower than those used in studies quantifying impairments by Δ^9^-THC content, refer to doses in Supplementary Table 1).

Working memory performance was also shown to be associated with tolerance effects: non-users made more errors during the Δ^9^-THC condition relative to placebo when compared to occasional users (53). Similarly, reduced accuracy and increased reaction times on attention tasks were observed only among occasional users relative to placebo, and not among regular/heavy users (52, 55, 56). Studies investigating how previous cannabis use modulates performance on response inhibition tasks showed inconsistent evidence (54–56). In summary, it appears that the most frequent users of cannabis develop a targeted tolerance to the most robust Δ^9^-THC effects on cognition (i.e., memory, working memory, and attention).

### Comorbidity With Psychosis-Spectrum Disorders

Considering that acute Δ^9^-THC exposure can induce transient positive psychotic symptoms among healthy individuals (30), and that cannabis-related cognitive deficits resemble the constellation of cognitive impairments observed in psychosis (57), this section focused exclusively on the modulating effect of a psychosis diagnosis or psychosis vulnerability in the relationship between cannabis and cognition. Results from robust between-subject comparison (patients vs. healthy controls) of cross-over placebo-controlled designs (within-subject design) do suggest an enhanced sensitivity to the cognitive impairing effect of Δ^9^-THC in psychosis (refer to Supplementary Table 4 for a summary of studies). For instance, D'Souza et al. (58) demonstrated that schizophrenia patients, relative to non-psychiatric individuals, showed greater verbal learning and verbal memory deficits following Δ^9^-THC administration relative to placebo. Another study revealed that adults with a genetic vulnerability to the psychosis-inducing properties of cannabis (Val/Val carriers on the catechol-O-methyltransferase (COMT) gene) were significantly more impaired on verbal and visual memory (not learning) following Δ^9^-THC exposure, relative to those with a low genetic vulnerability (Met/Met and Val/Met carriers) (59). However, these studies failed to observe other drug condition (Δ^9^-THC vs. placebo) by group (diagnosis or genetic vulnerability) interactions for attention performance and psychomotor speed (60, 61). Finally, in at least one study, negative results on the attention task seem to be driven by missing data and thus a low sample size (60). Convincing evidence from within-subject design revealed that a psychosis comorbidity may exacerbate the cognitive-impairing effects of cannabis, at the very least for memory.

## Residual Effects

### Cross-Sectional Studies

Residual effects refer to an array of measurable negative effects that persist after the state of intoxication. These residual effects have been assessed between ~12 h following cannabis exposure to more prolonged periods of abstinence (e.g., over 1 year). At least five meta-analyses including over 69 cross-sectional studies have collected data from more than 8,000 cannabis users and non-users who had undergone cognitive assessment (60–64). Worsened performances were consistently reported for learning and memory domains, with effect sizes ranging from small to moderate (60–64). Converging evidence from the meta-analyses also showed small deficits (Cohen's d ~0.2–0.3) in attention, executive functioning (i.e., inhibition and cognitive flexibility), and processing speed (refer to Table 2) (60–62). Interestingly, most of these domains (i.e., learning and memory, processing speed, and executive functions) were also more negatively affected in acute phases of intoxication, which suggests that adverse effects following cannabis intoxication persist days following cannabis abstinence. However, these cognitive deficits are categorized as mild. In comparison, residual effects of other substances, namely alcohol, cocaine and methamphetamine, are generally categorized as moderate (refer to Table 2) (65–67).

**Table 2:** Residual effects of cannabis use on cognitive functions in comparison to other substances.

**Cognitive domain**	**Substances**			
	**Cannabis effect size (*d*)**	**Alcohol effect size (*d*) (95% CI)**	**Cocaine effect size (*d*) (95% CI)**	**Methamphetamine effect size (*d*) (95% CI)**
Intelligence quotient	**–**	**−0.33 (−0.53**, **−0.13)**	**–**	**–**
Attention	**−0.36**	**−0.70 (−1.08**, **−0.32)**	**−0.59 (−0.87**, **−0.32)**	**−0.50 (−0.80**, **−0.20)**
Learning	**−0.35**	**−0.45 (−0.59**, **−0.32)**	**−0.55 (−0.74**, **−0.36)**	**−0.48 (−0.60**, **−0.37)**
Memory	**−0.25**	**−0.38 (−0.62**, **−0.15)**	**−0.56 (−0.77**, **−0.34)**	**−0.40 (−0.51**, **−0.28)**
Working memory	**–**	**−0.53 (−0.70**, **−0.36)**	**−0.52 (−0.74**, **−0.30)**	**−0.54 (−0.68**, **−0.40)**
Executive function	**−0.21**	**−0.53 (−0.63**, **−0.44)**	**−0.32 (−0.48**, **−0.16)**	**−0.45 (−0.55**, **−0.36)**
Processing speed	**−0.34**	**−0.47 (−0.58**, **−0.36)**	**−0.45 (−0.60**, **−0.29)**	**−0.37 (−0.49**, **−0.25)**
Visuospatial abilites (motor component)	**–**	**−0.49 (−0.62**, **−0.36)**	**−0.33 (−0.58**, **−0.08)**	−0.27 (−0.56, 0.01)
Verbal fluency	**−0.23**	**−0.40 (−0.54**, **−0.25)**	**−0.22 (−0.38**, **−0.06)**	**−0.43 (−0.65**, **−0.20)**

*CI, Confidence Interval*.

*Effects presented in bold are significant*.

*Data presented in this table represent effect sizes (Cohen's d) calculated from meta-analyses. Cannabis effect sizes represent the mean of effect sizes reported in the five meta-analyses investigating the residual cognitive effects (60–64); thus, the confidence interval is not reported. Alcohol, cocaine, and methamphetamine effect sizes come from the following meta-analyses: (65–67), respectively. Effect sizes are negative, which means that decreases in cognitive performances were observed in users relative to non-users. An effect size of ~−0.2 is considered as small; an effect size of ~−0.5 is considered as moderate; an effect size of −0.8 is considered as large. Among studies that investigated residual effects of cannabis use, cognitive assessments were done after a period that varied from many hours to 31 days (4 $\frac{1}{2}$ weeks). Similarly, the average abstinence period in studies focusing on alcohol was between 0 and 31 days. For studies focusing on cocaine, abstinence periods varied from a few days to 12 weeks. At last, the average abstinence periods for studies focusing on methamphetamine was 3.3 months*.

The aforementioned meta-analyses also investigated the potential moderating effect of covariates such as age of cannabis use onset, age of participants, duration of use, duration of abstinence, and frequency of use. There is converging evidence that neither age of cannabis initiation, age of participants (adolescents vs. adults), nor duration of use were significant moderators (60–64). The other two covariates are discussed in the following sections. Finally, in section Comorbidity with psychosis-spectrum disorders we discussed results from other meta-analyses which have focused on how psychosis spectrum comorbidity impacts the residual cognitive effects of cannabis use.

#### Duration of Abstinence

When meta-analyses focused on more chronic residual effects relative to effects from short abstinence periods, users (generally adults) no longer showed cognitive deficits, or showed significantly milder deficits. This finding was demonstrated by Scott et al. (62) for abstinence periods that persisted for more than 3 days, by Schoeler et al. (64) following 10 days of abstinence, and by Schreiner et al. (60) after ~1 month of cannabis use abstinence. This suggests that these residual effects have a short-term duration, but more importantly, that they are reversible. In the case of other substances like alcohol, cocaine and methamphetamine, residual effects that persisted after a month of abstinence (e.g., attention, learning, memory, and executive functioning) were instead categorized as moderate to large effect sizes. Before prematurely concluding that cannabis use is safer than other substance use, it should be noted that the majority of studies focusing on alcohol, cocaine and methamphetamine only included individuals who correspond to the Diagnostic and Statistical Manual of Mental Disorders (DSM) criteria for substance abuse, which complicates comparisons between various substances.

#### Deficits Increase as a Function of Use

When the effects of the frequency of cannabis use or a diagnostic of CUD are assessed on the amplitude of associated cognitive deficits, research showed a dose-response effect. Schoeler et al. (64) ascertained that mild use (e.g., <10 joints per month) was not associated with decreases in cognitive functioning; regular use (multiple times per week) was associated with deficits that were characterized as mild; and finally, daily use was associated with deficits that ranged from mild to moderate. Moreover, the cognitive deficits from daily use resembled alcohol-induced impairments in terms of importance, more specifically with regards to episodic memory. Similarly, individuals who are seeking treatment for substance abuse show global cognitive deficits of moderate amplitude, whereas those who do not seek treatment for substance abuse show only mild deficits (62). These moderate effect sizes for heavy cannabis users (criteria for abuse) resemble the severity of cognitive impairments reported in studies investigating the residual effects of other substances. Of note, the comparison between the residual cognitive effects of cannabis relative to other substances is challenging considering that the meta-analyses investigating alcohol, cocaine and methamphetamine included individuals meeting criteria for abuse and/or dependence (65–67), while the vast majority of studies on cannabis included a wide range of users (from light to heavy users) not meeting those criteria. With regards to the duration of cognitive deficits in regular and daily users, findings are difficult to interpret, given that they are controversial. That is, many authors report that cognitive deficits in intelligence quotient (IQ), attention and episodic memory (e.g., learning) that are associated with chronic (daily) cannabis use persist even 3 to 4 weeks following abstinence (68–70). However, other studies have also shown that these residual effects are reversed with >1 month of abstinence, and this was also the case for chronic users (71–74).

Altogether, residual effects of cannabis use can be observed on a myriad of cognitive abilities, such as learning and memory, executive functions, and processing speed. These deficits are generally less severe than those observed for alcohol, cocaine and methamphetamine and also seem to be reversed more quickly. However, effects of cannabis on memory (also possibly executive functioning and processing speed) are similar to those of alcohol and cocaine when frequency and severity of use are considered.

In the absence of experimental designs, studies evaluating the residual effects of cannabis are observational and usually utilize cross-sectional between-subject designs, in which users are compared to non-users matched on potential confounding variables. This type of research design does not allow for inferences on causality—that is, if the observed cognitive deficits were present or not before cannabis use and if they are not explained by other confounders. Consequently, the following section focused on longitudinal population-based and genetically-informed (co-twin designs) studies that better address these issues.

#### Comorbidity With Psychosis-Spectrum Disorders

Meta-analyses of cross-sectional studies do not provide support for hypothesis that individuals with psychosis are more sensitive to the residual effects of cannabis, in contrast to observations from acute challenge studies. To the contrary, two meta-analyses concluded that cannabis-using psychosis patients exhibited superior (small-to-moderate effects) cognitive functioning for attention, executive functions, working memory, delayed memory, verbal fluency, and visuo-spatial abilities relative to non-using patients (75, 76). A further meta-analysis of first-episode psychosis patients did not observe significant differences in neurocognitive performance between patients with and without cannabis use (77). It is important to interpret these results with caution. For example, studies that utilize a diagnosis of CUD as an inclusion criterion often include individuals with a current diagnosis alongside those with a history of CUD who are now in remission (75), therefore introducing noise to the data. Moreover, results that support higher cognitive function in cannabis-using patients do not extend to those with heavy use (daily) or CUD. In their large multi-country study, Ferraro et al. (78) confirmed that the higher IQ observed in cannabis-using patients relative to non-using patients was attributable to patients with occasional but not daily use. A recent exploratory analysis reported that among psychosis patients with CUD, greater cumulative cannabis exposure was associated with poorer performance across several cognitive domains (attention, working memory, delayed memory, decision making, and response inhibition) (79). The direct comparison of cognitive performance between cannabis users with and without co-morbid psychotic disorders provides further support for the hypothesis that individuals with psychosis are more sensitive to the cognition-impairing effects of heavy cannabis use. Following a 1-month abstinence period, significant improvements in verbal memory were observed for psychosis patients with CUD relative to non-psychiatric individuals with CUD while controlling for performance prior to abstinence (70). It was proposed that this greater recovery of memory function following abstinence reflects a greater vulnerability to its impairing effects in psychosis. Altogether, the available evidence suggests that individuals with psychotic disorders who are occasional (but not heavy) users of cannabis may represent a phenotypically distinct patient group with more intact (premorbid) cognitive functioning. Importantly, more severe patterns of cannabis use (e.g., CUD or daily use) eventually negatively interfere with cognitive performance; a finding that is in agreement with the literature on acute effects.

### Longitudinal Observational Studies

**Figure 1:**
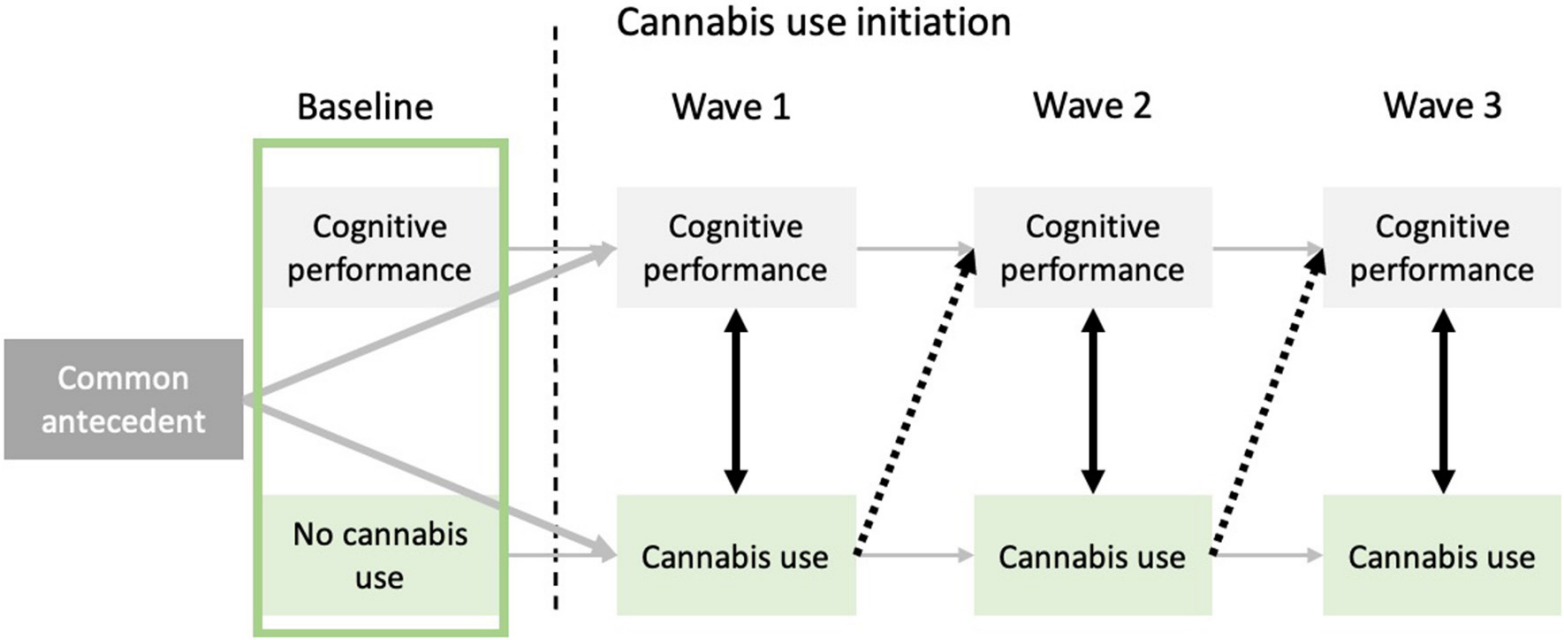
Representation of the cognitive vulnerability, concurrent, and neurotoxicity hypotheses relative to the association between cannabis use and cognitive functioning. The cognitive vulnerability hypothesis (represented by the green square) posits that before onset of cannabis use, future cannabis users already exhibit cognitive deficits. The common antecedent hypothesis, which offers a more general framework than the cognitive vulnerability hypothesis, posits that unknown common factors could be responsible for cannabis use onset and mild cognitive deteriorations, without cannabis use being the causal factor of the aforementioned cognitive deficits. Black dotted arrows allow to investigate the neurotoxic hypothesis by testing if previous cannabis use (*t*−1) predicts subsequent cognitive functioning (*t*), while controlling for frequency of cannabis use at time *t*. Lastly, black bidirectional arrows between cognitive abilities and cannabis use at every time-point represent the concurrent hypothesis. Indeed, cognitive performance at time *t* is associated with cannabis use at time *t*, without necessarily persisting effects through time.

Results from prospective designs may agree with three non-mutually exclusive hypotheses linking cannabis use and cognitive functioning. The *cognitive vulnerability* hypothesis postulates that cognitive deficits are already present before the onset of cannabis use for individuals who present higher risk of becoming regular users. This vulnerability hypothesis is often formulated within the more general common antecedent hypothesis. The latter proposes that common factors may predispose individuals to both cannabis use and mild cognitive decline in users, without cannabis use being the cause of these cognitive deficits, and without any specificity about the timing of such deficits. In contrast, the *concurrent* hypothesis posits that cannabis use is associated with cognitive deficits when controlling for premorbid cognitive performance, but only in short-term. It is proposed that abstinence or decreases in cannabis use should help alleviate these deficits. Lastly, the *neurotoxicity* hypothesis stipulates that past cannabis use induces a cognitive decline that persists even after individuals refrain from or decrease their cannabis use, when adjusting for cognitive functioning prior to cannabis use (see Figure 1 for a graphical representation of the three hypotheses within the context of mixed effects linear modeling).

#### Cognitive Vulnerability and Common Antecedent Hypotheses

The premorbid cognitive vulnerability hypothesis (e.g., before the onset of cannabis use) has been confirmed by recent studies. Findings show that future cannabis users already show lower performance at IQ tasks (non-verbal and verbal), memory, and executive functions (e.g., inhibitory control) when compared to individuals who remain non-users (80–84). As such, specific cognitive deficits seem to predispose individuals to earlier onset and more regular cannabis use. However, other studies did not provide evidence that cognitive impairment was apparent prior to cannabis use initiation (68, 85–88). As evidenced by rigorous co-twin designs, this cognitive vulnerability disappears when investigating individuals nested in a family, such that monozygotic and dizygotic twins discordant for cannabis use or cannabis dependence do not show differences in cognitive abilities prior to cannabis initiation (83, 84). These later twin studies do not support the purely cognitive vulnerability hypothesis, but do support the idea that common antecedents such as family factors (i.e., genetic and shared environment factors) explain this cognitive vulnerability observed at the population level. Clinical and behavioral factors have been put forth as common factors that predispose individuals to both cannabis use and cognitive deficits (89). For example, externalizing disorders as well as behavioral disinhibition have been positively associated with substance use and negatively associated with IQ (90, 91), suggesting that youths exhibiting externalizing symptoms and delinquency are less likely to be motivated to perform well at school and thus disengage from learning, and are more likely to use substances as a consequence of these problems.

#### Concurrent Hypothesis

When accounting for premorbid cognitive performance, cannabis use was associated with cognitive decline, at least in the short-term (during the same assessment intervals), in executive functioning, general IQ, memory, processing speed, and visuospatial abilities in several studies (68, 71, 81, 85, 88, 92, 93). Declines in cognitive functioning were observed years after the onset of cannabis use and were obvious even when taking into account other substance use (68, 71, 81, 85, 93), academic achievement (68, 85, 92), externalizing problems or other mental health comorbidity (68, 71), and socioeconomic status (71, 81, 85, 88, 94). Without eliminating the possibility that these factors could have played a mitigating role, controlling for these covariates increases our confidence in the idea that cannabis could have deleterious effects on cognitive functioning. Only a few studies did not report concurrent impairing effects of cannabis use (82, 86). Of note, among the studies that investigated the concurrent hypothesis from a within-subject perspective, two out of three revealed that if an individual shows increases in cannabis use frequency at a given assessment, they will also show lower executive functions performance during that same assessment period (80–82). The results were partially replicated within co-twin designs. Among several tests measuring non-verbal and verbal IQ, as well as executive functioning (i.e., working memory, response inhibition, and cognitive flexibility), poorer performance in twins who used cannabis more frequently than their co-twin was limited to two tasks (one measuring working memory, the other, non-verbal IQ) (83, 84, 95). Altogether, these findings are in line with impairments in cognitive domains that were underlined by meta-analyses of cross-sectional studies investigating residual effects of cannabis use, as well as studies focusing on the acute effects of Δ^9^-THC intoxication.

#### Neurotoxic Hypothesis

Longitudinal studies provide mixed evidence for the neurotoxic hypothesis. On the one hand, former regular users showed better cognitive development than current regular users (92) and even performed as well as non-users (71), suggesting that cannabis impairing effects tend to resolve following abstinence. Similarly, Jacobus et al. (93) demonstrated that cannabis users performed more poorly than non-users across various cognitive domains, yet this performance difference disappeared at the last follow-up when users had reduced their overall consumption. On the other hand, cannabis use frequency was shown to predict subsequent cognitive decline in executing functioning and verbal intelligence regardless of whether cannabis use continued (87, 88). Specifically, Castellanos-Ryan et al. (80) and Meier et al. (68) provided evidence that a significant reduction of cannabis use (from daily to light user) or abstinence in the 12 months prior to cognitive testing were still significantly associated with a decline in executive functioning and general IQ. Furthermore, in their population cohort, Morin et al. (81) observed that over and above the concurrent impairing effect of cannabis use at the individual level, if one increases their cannabis use frequency in a given year, one will also show lower performance on response inhibition a year later. This latter study provides robust evidence of a long-term (at least 12 months) or neurotoxic effect of cannabis use considering that individuals who changed their patterns of cannabis use through the follow-ups were compared to themselves. Despite these proofs of neurotoxic effects from cannabis use with extensive covariate control, we cannot rule out the possibility that part of the variance between cannabis and subsequent poorer cognitive performance comes from indirect causal effects, for example, through social milieu (96, 97).

#### Factors Modulating the Residual Cognitive Effects of Cannabis

##### Quantities Used

In line with cross-sectional studies, it is when we distinguish occasional, regular and heavy users that cognitive deficits in memory or processing speed become more apparent (71). Indeed, memory deficits associated with weekly use of cannabis are in the range of moderate effect sizes (98), which bears resemblance to the effects of alcohol abuse. Similarly, other findings show that for each 5-year period of cannabis use, performance on memory tasks progressively decrease (99). Beyond long-term memory, research has shown that frequency and dependence of cannabis use are positively related to worse executive function and IQ deficits (68, 80, 81, 84, 85, 87). A paucity of studies did not report dose-response effects on associated cognitive deficits (82, 83, 86, 100, 101) however, some of these studies assessed cognitive domains that are not considered to be affected by cannabis use (e.g., lexical knowledge) (83, 101).

##### Cognitive Domains

It is important to underline that not all longitudinal studies have assessed residual effects of cannabis use on cognitive functioning more broadly. For example, a few studies have focused solely on the association between cannabis use and verbal fluency (88) or orientation [Mini Mental State Examination: (101)], and have therefore not reported any associations between cannabis use and cognitive deficits. When considered alone, these studies may falsely lead us to believe that cannabis use does not alter cognitive performance, regardless of the studied cognitive domain. However, converging findings from all studies help better explain the relation between cannabis use and cognitive deficits. Indeed, among 10 prospective studies that assessed memory, eight reported specific deficits in this cognitive domain (71, 74, 80–82, 92, 93, 98–100). Likewise, 7 of 10 studies investigating associations between cannabis use and executive function (i.e., response inhibition) showed declines in performance linked to cannabis use (68, 80–82, 84, 87, 93, 95, 99, 100). Findings of effect on processing speed, however, are less robust with three of seven studies reporting declines in performance linked to cannabis use (68, 71, 82, 92, 93, 98, 99). Finally, long-term effects of cannabis use on non-verbal IQ are mildly probable, as 6 of 10 studies have failed to show significant associations here (68, 71, 81–86, 93, 95).

##### Age of Cannabis Initiation

An increasing number of studies have endeavored to test the hypothesis that adolescence consists in a vulnerable period to the impairing effects of cannabis use. Generally, results can be summarized as follows (i) for an equivalent consumption, cognitive deficits seem to be more important in those who initiated cannabis use younger (e.g., during adolescence) (68), (ii) deficits noted in adolescents are similar to those observed in adults, but appear following less intensive use of cannabis (80, 81, 87); (iii) a combination of both. For example, an interesting study showed potentially additive negative effects on global performance on IQ tasks between the number of years of cannabis use and age of onset that is earlier than 18 years old (68). Moreover, the dose-response relationship highlighted by Meier et al. (68) on IQ performance was explained by adolescent-onset cannabis use, not adult-onset use. Studies conducted on three independent samples of Canadian and US adolescents have shown that increases in cannabis use during high school predicted cognitive declines in performance on memory and executive functions tasks a few years after assessment (80, 81, 87). In addition to this, it should be noted that these cognitive effects were noted in young individuals who were for the most part not heavy users (<weekly use). Moreover, age of onset of cannabis use that was prior to 15 years old compared to age of onset that occurred after 14 years old was related to impaired development of inhibition capacities, independently of the frequency of cannabis use (80). Critically, these deficits seemed more permanent than the ones reported by adults (71, 98). That is, increases in cannabis use during adolescence were associated with declines in executive functioning and IQ scores at age 20, and even until age 38, and this was also the case for individuals who had considerably reduced their consumption 12 months prior to cognitive assessments (68, 80). Taken together, these findings suggest that adolescence represents a critical period for vulnerability to deleterious effects of cannabis use on cognitive functioning.

## Discussion

The current comprehensive review highlights that the acute administration of cannabis/THC produces moderate impairments in episodic and working memory, as well as small to moderate deficits in processing speed and executive functions. Impairments in attention and impulsivity have also been documented but are smaller. In the case of speed of processing, there is evidence showing that the impairments are less severe in oral administration studies relative to studies using other routes of administration (e.g., smoked, inhaled, injected). Although some studies have shown that higher Δ^9^-THC concentrations are associated with more prominent cognitive impairments, further studies are required to establish what doses are problematic. Likewise, there is preliminary evidence showing the cannabidiol may attenuate Δ^9^-THC-induced cognitive impairments, but results are inconclusive thus far. While several studies on the acute effects of cannabis/Δ^9^-THC have paid attention to traditional cognitive domains such as attention, episodic memory, executive functions, speed of processing, and working memory, there is a relative lack of research on the effects of cannabis/Δ^9^-THC on social cognition (e.g., theory of mind and emotion recognition).

Cross-sectional studies on the residual cognitive effects have generally shown that cannabis is associated with cognitive deficits that are relatively small and seem to abate after a relatively short period of abstinence. Such studies seem to indicate that cannabis produces smaller cognitive deficits than those produced by alcohol, cocaine or methamphetamine, which typically produce moderate deficits in several cognitive domains. It is crucial to point out, however, that the meta-analyses on alcohol, cocaine and methamphetamines have been performed using studies involving individuals with a substance use disorder, whereas the great majority of studies on cannabis have been performed in occasional, regular or frequent users. Future studies in the field will need to pay attention to individuals meeting the criteria for a cannabis use disorder.

Due to the methodological limitations of cross-sectional studies, a growing number of high-quality longitudinal studies have been performed in recent years. In these studies, residual impairments were observed mostly in the same cognitive domains (e.g., verbal learning and memory, speed of processing) that have been shown to be impaired in the acute administration studies. Research results suggest that the cognitive effects following cannabis intoxication persist at least days or weeks following cannabis abstinence in regular users. Relative to adult-onset, adolescent-onset cannabis use seems to explain the dose-response relationship that has been observed and is associated with longer lasting residual effects even in not so heavy users (<weekly). The association between cannabis and cognition is likely explained by common antecedents, such as genetics and shared environment factors. To a lesser degree, cannabis may also produce neurotoxic effects. Further large-scale longitudinal studies on the cognitive effects of cannabis are required, paying careful attention to premorbid cognitive performance, dose-response, cannabis constituents, and potential common antecedents.

As for the cognitive effects of cannabis in individuals with a comorbid psychiatric disorder, such as schizophrenia, research results are unfortunately difficult to interpret as the vast majority of studies in the field have adopted cross-sectional designs. Clearly, longitudinal studies in these populations are warranted. Finally, it is worth mentioning that the literature on “synthetic cannabinoids” is scarce. Considering that “synthetic cannabinoids” are full agonists at CB_1_ receptors (in comparison, Δ^9^-THC is a partial agonist), they may theoretically produce cognitive impairments that are more prominent and longer lasting than those of cannabis (102). With a growing number of states and countries liberalizing their policies on cannabis, the study of the cognitive effects of cannabis has important implications, since cannabis smoking may be associated with lower academic achievement, decreased work performance, and increased rates of motor vehicle accidents. Careful attention will need to be paid to policies and program that could minimize these undesirable outcomes. Such measures include disseminating public health campaigns on the hazards of cannabis use, implementing evidence-based preventive interventions in schools, prohibiting the marketing of cannabis products in ways that are attractive to youth, taxing cannabis products based on their Δ^9^-THC content, and regulating maximal Δ^9^-THC concentrations.

## Author Contributions

JB and SP reviewed the literature. JB wrote the manuscript. SP provided critical comments.

## Conflict of Interest

The authors declare that the research was conducted in the absence of any commercial or financial relationships that could be construed as a potential conflict of interest.
