# Supplementary material for: Cannabis and Cognitive Functioning: From Acute to Residual Effects, From Randomized Controlled Trials to Prospective Designs
Source: Front Psychiatry. 2021 Jun 10;12:596601. doi: 10.3389/fpsyt.2021.596601 (PMC8222623; doi:10.3389/fpsyt.2021.596601)
Supplement: Supplementary file 1 [file Data_Sheet_1.docx]

Supplementary Online Content for Bourque et al., Cannabis and Cognitive Functioning: From Acute to Residual Effects, From Randomized Controlled Trials to Prospective Designs. Frontiers in Psychiatry

**Content**

**Table S1.** Randomized controlled trials investigating the acute effects of cannabis use on cognitive functioning by dose of Δ-9-THC.

**Table S2.** Studies investigating the acute effects of cannabis use on cognitive functioning by Δ-9-THC:CBD ratio.

**Table S3.** Studies investigating the acute effects of cannabis use on cognitive functioning by psychosis spectrum comorbidity.

**Table S4.** Longitudinal observational studies and quasi-experimental co-twin studies of the association between the residual effects of cannabis use on cognitive functioning.

**References**

**Table S1.** Randomized controlled trials investigating the acute effects of cannabis use on cognitive functioning by dose of Δ-9-THC.

| Author | Design | Doses | Participants | Cognitive domains | Findings | Analytical strategy |
| --- | --- | --- | --- | --- | --- | --- |
| D’Souza et al.  2004^1^ | Cross-over (within individuals) | Intravenous 0, 2.5, and 5.0mg of Δ-9-THC | 22 not cannabis-naive (no lifetime criteria for CUD) | Verbal learning and memory, vigilance and distractibility, verbal fluency, working memory | Δ-9-THC significantly impaired immediate and delayed recall in a dose-dependent manner. Δ-9-THC had significant dose effects on omission errors, but not commission errors for the distractibility task. No dose-dependent effect for vigilance, verbal fluency and mixed findings for working memory. | Non-parametric rank-based analysis |
| Curran et al.  2002^2^ | Cross-over (within individuals) | Oral capsules of 0, 7.5, and 15mg of Δ-9-THC | 15 infrequent male users. Current weekly cannabis users were excluded. | Verbal learning and memory, attention/working memory, executive function, working memory, reaction time, focused attention, implicit memory, verbal fluency | There were dose-dependent impairments by THC for verbal memory, but not verbal learning. Also, both doses of THC were associated with poorer performance on focused attention, however there was no explicit information about the presence or absence of a dose-response relationship. For verbal fluency and immediate and delayed recall domains, only the higher dose negatively affected performance. Finally, reaction times and performance on attention/working memory, implicit memory, and executive function tasks were not impaired by neither THC doses. | Repeated measure multivariate analyses of variance with both drug condition (3 doses) and time as within-subject variables |
| Hunault et al.  2009^3^ | Cross-over (within individuals) | Smoked cigarettes containing 0, 29.3, 49.1, and 69.4mg of Δ-9-THC | 24 non-daily male users | Reaction time, visuo-spatial selective attention, short-term memory, divided attention, and sustained attention | Response time (RT) increased significantly with increasing THC doses which means a linear dose–effect relationship was observed for RT among all tasks. The average number of errors differed significantly between the four THC doses in the short-term memory and the sustained attention tasks (linear dose-effects) but not in the selective attention task nor in the short-term memory part of the divided attention task. Analyses were conservative because only linear dose-response relationships were investigated. | Multilevel model (observations clustered within individuals) with dose as continuous variable |
| Weinstein et al.  2008^4^ | Cross-over (within individuals) | Smoked cigarettes containing 13 and 17mg of Δ-9-THC | 14 daily users (10 males) | Cognitive flexibility, decision making | For cognitive flexibility, there was no difference in the total number of blocks completed without errors as well as non-perseverance errors between the session at 13mg and at 17mg. No dose effect for speed of processing and quality of decision making in the decision making task. | ANOVA |
| Hart et al.  2001^5^ | Cross-over (within individuals) | Smoked cigarettes containing 0%, 1.8%, and 3.9% Δ-9-THC concentrations | 18 almost daily users (6.1 days/week; 10 males) | Reaction time, attention, working memory, learning and delayed recall, visuospatial processing, reasoning, cognitive flexibility and mental calculation | In the 3.9% concentration condition, participants took more time to finish completing immediate recall and mental calculation tasks relative to the 1.8% condition.  No significant differences were observed when accuracy of responding on attention, visuospatial processing, reasoning, cognitive flexibility, and mental calculation tasks were analyzed as a function of Δ^9^-THC concentration. Among the memory tasks, only performance on immediate recall was impaired following administration of the highest concentration of Δ-9-THC relative to 0% and 1.8% cigarettes. | Repeated measures ANOVA |
| Ramaekers et al.  2006^6^ | Cross-over (within individuals) | 0, 250, and 500 μg/kg of smoked Δ-9-THC | 20 light users (average of 3 times/monthly, 14 males) | Response inhibition, decision making | Relative to placebo, Δ^9^-THC reduced the number of correct decisions and increased stop reaction time in the response inhibition task.  The proportion of observations showing impairment progressively increased as a function of serum Δ^9^-THC in every task. At concentrations between 5 and 10 ng/ml approximately 75-90% of the observations were indicative of significant impairment in every performance test. At Δ^9^-THC concentrations >30 ng/ml the proportion of observations indicative of significant impairment increased to a full 100% in every performance test. | -Repeated measures MANOVA with THC (3 doses) as a main factor.  -Linear regression analysis between THC concentrations in serum and oral fluid and changes (from placebo) in task performance |
| Spindle et al. 2018^7^ | Cross-over (within individuals) | 0, 10, and 25mg Δ-9-THC doses were vaporized and smoked | 17 infrequent users (had not used in the previous month) | Selective and divided attention, working memory | Did not explicitly test for dose-response relationships, but the authors reported that the magnitude of performance impairment for the attention and working memory tasks increase with higher doses - more so for the vaporized conditions. | Repeated-measure regressions with 3 factors included: time (change from baseline scores), dose (0 mg, 10 mg, and 25 mg), and inhalation method (smoked vs vaporized) |
| Adam et al. 2020^8^ | Cross-over (within individuals) | Experiment 2: Oral capsules of 0, 7.5, and 15mg Δ-9-THC | 23 occasional users (non-daily use; 11 males) | Visual working memory | Although polynomial contrasts revealed a linear effect of dose on working memory performance, only the high dose was significantly different from placebo. | One-way repeated-measures ANOVA with dose as a factor (placebo, 7.5mg and 15mg). |
| Heishman et al. 1997^9^ | Cross-over (within individuals) | Smoking 4, 8, and 16 puffs of 3.55% Δ-9-THC, and placebo (in every condition participants had to smoke 16 puffs) | 5 regular male users (1-6 marijuana joint/week) | Reaction time, processing speed/attention, working memory, verbal learning | The 8- and 16-puff cannabis conditions impaired performance on the processing speed/attention task, but not in an orderly dose-related manner. There were no significant cannabis condition or condition × time effects on reaction time and working memory tests. A cannabis dose-related decrease in the number of words recalled (verbal learning) after each list presentation was observed (revealed by a significant presentation order × drug condition interaction). | Repeated-measures ANOVA with dose (4, 8, and 16 puffs) and time (mid-smoking, post-smoking) as factors. |
| Böcker et al.  2010^10^ | Cross-over (within individuals) | Joints containing 29.3mg, 49.2mg, and 69.4mg of Δ-9-THC, and a placebo joint with less than 0.003% c | 24 occasional male users (2-18 joint/month) | Selective attention | A significant linear dose-related effect was observed between dose and speed (reaction time) and accuracy, such that mean reaction time and accuracy were lowest in the placebo condition and highest in the high dose condition. | Repeated-measures MANOVA with a dose factor (placebo, low, medium, and high). |

**Table S2.** Studies investigating the acute effects of cannabis use on cognitive functioning by Δ-9-THC:CBD ratio.

| Author | Design | Conditions | Participants | Cognitive domains | Findings | Analytical strategy |
| --- | --- | --- | --- | --- | --- | --- |
| Hindocha et al.  2015^11^* | Cross-over (within individuals) | Vaporized THC only (8mg), CBD only (16mg), THC+CBD (8mg + 16mg), and placebo | 24 light (<24 days/month) and 24 heavy (>25 days/month) users (34 males) | Emotion processing | Collapsing over emotion types, participants showed poorer accuracy after THC alone compared to placebo at the 40% facial expression intensity. Subsequent paired-samples *t*-tests revealed that participants were more accurate in the THC+CBD condition relative to the THC alone condition. | Mixed model repeated-measures ANOVA with drug condition, emotion and intensity (of facial expression) as within-subject factors. |
| Schoedel et al.  2011^12^ | Cross-over (within individuals) | 6 conditions: Oromucosal spray of 10.8, 21.6 and 43.2mg Nabiximols (1:1 THC:CBD), and 20 and 40mg Dronabinol (synthetic THC without CBD), and placebo | 23 frequent users (> once/week in the previous 3 months, 19 males) | Reaction time, divided attention, working memory  *Cognitive testing was done over 24h post-dose | The drug conditions had no effect on reaction times and performance on the divided attention test. Only the high dose of synthetic THC (dronabinol) had increased reaction time during the working memory task compared with placebo, while the lower dose of dronabinol and all doses of nabiximols (THC:CBD ratio of 1) were not different from placebo. | Linear mixed-effect models with drug condition sequence, period, drug condition as fixed effects, baseline information as covariate, and subject within the drug condition sequence as a random effect |
| Morgan et al.  2018^13^* | Cross-over (within individuals) | Vaporized THC only (8mg), CBD only (16mg), THC+CBD (8mg + 16mg), and placebo | 24 light (<24 days/month) and 24 heavy (>25 days/month) users (34 males) | Immediate and delayed recall, spatial working memory, verbal fluency, processing speed. | Both THC and THC + CBD conditions impaired performance on immediate and delayed recall as well as working memory tasks while performance during the CBD alone condition was not significantly different than placebo. For verbal fluency and processing speed, the THC and THC + CBD conditions did not impair performance relative to placebo. | Repeated measures ANOVA with drug condition as a within-subject factor |
| Arkell et al.  2019^14^ | Cross-over (within individuals) | 3 conditions: Vaporized 125 mg THC (11% THC, < 1% CBD), THC/CBD (11% THC, 11% CBD), or placebo (< 1% THC, < 1% CBD) | 14 occasional users (<2 times/week in the previous 3 months and >10 lifetime exposures, 11 males) | Processing speed/attention, divided attention, sustained/divided attention | Vaporized THC alone, but not THC combined with CBD, reduced processing speed/attention performance relative to placebo. For both the divided and sustained attention tests, participants showed impaired performance following THC combined with CBD compared to THC alone or placebo. | Linear mixed models with drug condition, time, session and drug condition by time as fixed factors. Covariates included baseline cognitive data |
| Lawn et al. 2016^15^ | Cross-over (within individuals) | 3 conditions: Vaporized THC (8mg), THC/CBD (8mg THC, 10mg CBD), and placebo | 17 occasional users (<3 times/week, 8 males) | Effort-related decision making | Administration of THC alone reduced the overall likelihood of making high-effort choices for monetary reward compared with placebo. This effect was not, overall, attenuated during the THC + CBD condition. | Generalized estimation equation models to predict likelihood of making a high-effort choice |
| Roser et al. 2009^16^ | Cross-over (within individuals) | 3 conditions: Oral capsules of 10mg Δ-9-THC, 10mg Δ-9-THC + 5.4mg CBD, or placebo | 24 occasional users (12 males, all right-handed) | Psychomotor speed | Administration of THC + CBD reduced right-hand tapping frequencies in all parts of test. In contrast to the THC + CBD condition, the THC condition had no marked effect on tapping test parameters compared to placebo. | MANOVA |
| Morgan et al. 2010^17^ | Naturalistic (between individuals) | Low CBD group: 22 users smoking strains with < 0.14% CBD  High CBD group: 22 users smoking strains with > 0.75% CBD | Occasional users (> once/month for 12 months) | Immediate and delayed recall, verbal fluency, episodic memory | Poorer performance was observed in the low CBD group relative to the high CBD group in both immediate and delayed recall, despite there being no between-group difference in the cannabis’ THC content and in performance while not intoxicated. There were no group differences on performance scores of the verbal fluency and episodic memory tasks | 2 x 2 repeated-measures ANOVA with group as a between-individual factor and day (intoxicated, not intoxicated) as a within-subject factor |
| Englun et al.  2013^18^ | RCT without cross-over (between individuals) | Group 1 (n=26): Pretreatment of oral capsules of placebo followed by intravenous THC (1.5mg)  Group 2 (n=22): Pretreatment of oral capsules of 600mg CBD followed by intravenous THC | Occasional users (27 males) | Verbal learning and memory, processing speed, working memory, executive functioning | Delayed but not immediate recall was poorer, relative to baseline, in the placebo pre-treated group compared with the CBD pre-treated group. No effect of group on processing speed, working memory and executive functioning. | Repeated-measures ANOVA with moment (baseline, post-capsule, post-THC) as a within-subject factor and group as a between-subject factor |
| Morgan et al. 2012^19^ | Naturalistic (between individuals) | Following CBD hair analysis, participant were further divided in a CBD absent and CBD present groups | 120 participants: 54 recreational users, and 66 daily users | Immediate and delayed memory, episodic memory | A significant main effect of CBD presence was observed only in the episodic memory task: individuals (across recreational and daily users) who had CBD present in their hair performed better than to those who did not have CBD present (across high and low THC levels). | 2x2x2 ANOVA with factors of group users (recreational, daily), CBD (present or absent) and THC level (high or low), with IQ as a covariate |

*Same sample and same design

**Table S3.** Studies investigating the acute effects of cannabis use on cognitive functioning by previous cannabis use.

| Author | Design | Conditions | Participants | Cognitive domains | Findings | Analytical strategy |
| --- | --- | --- | --- | --- | --- | --- |
| Colizzi et al.  2018^20^ | Between subject (non-users vs users) contrast of a cross-over (placebo vs THC) RCT | Oral capsule containing 10mg Δ-9-THC and placebo | 24 male participants:  12 non-users (<5 lifetime joints) and 12 occasional users (24.5±9 lifetime joints) | Verbal learning and memory, response inhibition  (tasks during fMRI session) | Performance on the verbal learning and memory task during the drug condition (while intoxicated) did not differ between non-users and occasional users (in the whole sample, there was no significant effect from THC on performance). Conversely, relative to placebo, the THC-induced increase in inhibition errors was greater in non-users than occasional users. | ANOVA with drug condition (THC, placebo) as a within-subject factor and user status (non-user, occasional user) as a between-subject factor |
| Ramaekers et al.  2009^21^* | Between subject (non-users vs users) contrast of a cross-over (placebo vs THC) RCT | Smoked cigarettes containing 13% of Δ-9-THC (for a total of 500μg/kg), and placebo | 24 participants:  12 occasional users (< once/week; 8 males) and 12 regular users (>4 days/week; 9 males) | Psychomotor function/attention, divided attention, response inhibition, and executive function (decision making) | The overall impairing effect of THC on psychomotor function/attention and divided attention performance was evident in occasional users relative to regular users. While performance on response inhibition was affected by THC, there was no specific effect of cannabis use status. Lastly, performance on the decision making task was not affected by THC. | Repeated-measure ANOVA with drug condition (THC, placebo), time of testing (4 levels) as within-subject factors and cannabis use status as between-subject factor |
| Theunissen et al.  2012^22^* | Between subject (non-users vs users) contrast of a cross-over (placebo vs THC) RCT | Smoked cigarettes containing 13% of Δ-9-THC (for a total of 500μg/kg), and placebo | 24 participants:  12 occasional users (< once/week; 8 males) and 12 regular users (>4 days/week; 9 males) | Divided attention, response inhibition | The impairing effect of THC on divided attention performance were specific for the occasional users. Only stopping reaction times were increased by the THC condition. There were no effect of cannabis use status or interaction with drug condition on the response inhibition task. | ANOVA with drug condition (THC, placebo) as a within-subject factor and cannabis use status as a between-subject factor |
| D’Souza et al.  2008^23^ | Between subject (non-users vs users) contrast of an RCT | 2 conditions: pretreatment with either placebo or haloperidol followed by intravenous placebo, and lastly followed by intravenous Δ-9-THC (0.0286mg/kg) | 11 occasional users (lifetime exposure >100 times and recent exposure > 10 times/month) and 17 non-users | Verbal learning and memory, attention (vigilance and distractibility), spatial working visual memory, executive function | For verbal learning, the combined effect of haloperidol and THC produced significant performance deficits in non-users, but not in occasional users. There were no main effects or interaction with cannabis use status on other measures of verbal memory, visual memory, attention and executive function. For spatial working memory, non-users reported increased THC-induced errors relative to occasional users. | Linear mixed models with haloperidol (placebo, active) and THC (placebo, active) as within-subject factors and cannabis use status as a between-subject factor |
| D’Souza et al. 2008^24^ | Between subject (non-users vs users) contrast of a cross-over (placebo vs THC) RCT | 3 conditions:  Intravenous 0, 2.5 and 5mg of Δ-9-THC | 30 regular users (met criteria for current cannabis use disorder; 21 males) and  22 non-users (<1 exposure in the past month; 14 males) | Verbal learning and memory, attention (vigilance and distractibility) | For verbal learning, regular users showed blunted THC-induced impairments relative to non-users. Similarly, for free delayed recall, only in non-users did THC impaired performance in a linear dose-dependent fashion. This impairing effect was not observed during delayed cue recall, not during delayed recognition recall. THC decreased distractibility performance only in non-users while it impaired vigilance performance specifically in regular users. | Non-parametric mixed model with dose (3 levels) as a within-subject factor and cannabis use status as a between-subject factor, and age and IQ as covariates. |

*Same sample and same design

**Table S4.** Studies investigating the acute effects of cannabis use on cognitive functioning by psychosis spectrum comorbidity.

| Author | Design | Conditions | Participants | Cognitive domains | Findings | Analytical strategy |
| --- | --- | --- | --- | --- | --- | --- |
| D’Souza et al.  2005^25^ | Between subject (patients vs healthy controls) contrast of a cross-over (placebo vs THC) RCT | 3 conditions:  Intravenous 0, 2.5 and 5mg of Δ-9-THC | 13 antipsychotic treated schizophrenia patients (10 males)  and 22 occasional healthy users (14 males) | Verbal learning and memory, vigilance and distractibility, verbal fluency | While accounting for the worse performance from schizophrenia patients on verbal learning at baseline (before intoxication), schizophrenia patients were more sensitive to the dose-related impairing effects of Δ-9-THC for verbal learning. As for verbal memory, schizophrenia patients performed generally worse than healthy controls, but the authors did not find any significant dose (THC vs placebo) x group interaction, suggesting no further susceptibility to Δ-9-THC memory impairment from patients. Similarly, for attention, there were no significant dose x group interactive effects, however low sample size (n=7) for this task specifically might explain this negative result. Finally, Δ-9-THC had no effect on verbal fluency performance in the whole sample. | Non-parametric rank-based analysis with group (patients vs controls) as a between-subject factor and dose as a within-subject factor |
| Henquet et al.  2006^26^ | Between subject (patients vs healthy controls) contrast of a cross-over (placebo vs THC) RCT | Smoked cigarettes containing 13% of Δ-9-THC (for a total of 300μg/kg), and placebo | 30 patients with psychosis, 12 first- and second-degree relatives of patients, and 32 healthy controls | Verbal and nonverbal learning and memory, attention (sustained and selective), psychomotor speed | The overall impairing effect of THC on psychomotor function/attention and divided attention performance was evident in occasional users relative to regular users. While performance on response inhibition was affected by THC, there was no specific effect of cannabis use status. Lastly, performance on the decision making task was not affected by THC. | Multilevel random regression analyses examining the effects of drug condition (Δ-9-THC vs placebo), genetic vulnerability (Val/Val, Val/Met, Met/Met), and clinical vulnerability. |

**Table S5.** Longitudinal observational studies and quasi-experimental co-twin studies of the association between the residual effects of cannabis use on cognitive functioning.

| Author | Sample size | Baseline age or age range | Length of follow-up | Assessments | Frequency of use | Cognitive domains tested | Cognitive vulnerability hypothesis | Concurrent hypothesis | Neurotoxic hypothesis | Analytical strategy | Model’s covariates |
| --- | --- | --- | --- | --- | --- | --- | --- | --- | --- | --- | --- |
| Auer et al.  2016^27^ | 3385 | 18-30 | 25 y | Cannabis measured at baseline, 2, 5, 7, 10, 15, 20, and 25 years follow-up: a cumulative lifetime use was estimated. Cognition measured at the 25 years follow-up. | 16% never used cannabis, 44% used cannabis daily for <0.5 years, 24% used cannabis daily for 0.5-2 years, 7% used cannabis for 2-5 years, and 9% used cannabis daily for >5 years. | Verbal memory, processing speed, executive function (response inhibition) | Could not test if cognitive impairment was present prior to cannabis use because participants had already initiated cannabis when entering the study | “Cumulative lifetime exposure to cannabis was significantly associated with worse verbal memory.” | Did not investigate whether cannabis impairing effects were present at a later follow-up regardless of whether cannabis use continues | Linear regressions | Tobacco, use of alcohol and illicit drugs, physical activity, depression. |
| Becker et al.  2018^28^ | 73 | 19-20 | 2 y | Cannabis measured at baseline. Cognition measured at baseline and follow-up (2 years later) | 38 cannabis users (90% reported using >5 times/week) and 35non-users (<once/monthly) | Motor function, attention, verbal fluency, verbal working memory, verbal learning and memory, spatial working memory, planning, and motivated decision making. | Could not test if cognitive impairment was present prior to cannabis use because participants had already initiated cannabis when entering the study | Groups by time interaction were observed for spatial working memory and decision making, such that cannabis users showed poorer performance than non-users at baseline, but not at follow-up. A later age of cannabis use onset was associated with better performance on verbal memory and planning relative to early onset users. | Did not investigate whether cannabis impairing effects were present at a later follow-up regardless of whether cannabis use continues | Repeated-measures ANOVA | Baseline IQ, sex, time interval between assessments, and alcohol use. |
| Boccio & Beaver.  2017^29^ | 373 to 6584 | 12-21 | 6 y (first follow-up 1 year later, and last follow-up 6 years later) | Cognition was assessed at baseline and wave 3 (6 years later). Cannabis was assessed at wave 2 (1 year later) and wave 3 (6 years later) | Ever user vs never users. 12% had used cannabis in the previous year at wave 2, while 70% had used cannabis in the previous year at wave 3. | Verbal intelligence | No evidence that cognitive impairment was apparent prior to cannabis use initiation. | Having tried cannabis at wave 3 was associated with a decline in verbal intelligence performance between waves 1 and 3, relative to non-users (modest effect size). | Having tried cannabis at wave 2 predicted a significant decline in verbal intelligence performance from waves 1 to 3, relative to non-users (modest effect size). | OLS regression with IQ change score as the dependent variable | Age, sex, race, SES, and personality factors. |
| Castellanos-Ryan et al.  2017^30^ | 294 | 13 | 7 y | Cognition measured at ages 13, 14, 20. Cannabis measured annually between ages 13 and 17. | Prevalence of cannabis use ranged from 43% at some point during adolescence to 51% at age 20 (last assessment) | Verbal IQ, memory, working memory, executive functioning, response perseveration. | Poor memory and working memory performance were associated with earlier onset of cannabis use. Higher verbal IQ was associated with both earlier onset and a steeper increase in cannabis use frequency. | No concurrent effects examined. | Early cannabis use frequency (age 14) was significantly associated with a performance decline (from 13-20 years old) on response perseveration. The steeper the growth in cannabis use from 14-17 years old was significantly associated with poorer development of executive functioning. In those who had abstained from using cannabis 12 months prior to cannabis testing at 20 years, the increase in cannabis use across adolescence (slope) was still significantly associated with a decline in executive functioning from preadolescence to early adulthood. | Latent growth curve modelling using cognitive change scores | SES, academic achievement, externalizing problems, alcohol use. |
| Fried et al.  2002^31^ | 70 | 9-12 | 8 y | Cognition measured prior to cannabis use (9-12 y.o.) and during young adulthood (17-20 y.o.). Cannabis measured at 17-20 y.o. | 4 groups: 37 non-users (never used marijuana weekly); 9 former regular users; 9 light current users (<5 joints/week); and 15 heavy current users (>5 joints/week). | General IQ | No difference in preteen IQ across the 4 groups. | IQ difference score for the heavy current user group was significantly different from that for non-users. | Did not investigate whether cannabis impairing effects were present at a later follow-up regardless of whether cannabis use continues, but did report no significant differences between former users and non-users. | ANCOVA with IQ differences score as dependent variable. | SES, education, age, sex, other substance use, maternal use of substances during pregnancy, and mother's age at time of birth. |
| Fried et al.  2005^32^ | 113 | 9-12 | 8 y | Cannabis use measured at last follow-up. Cognition measured at ages 7, 9-12, 13-16, and 17-21. | 4 groups: 19 current light users (<5 joints/week), 19 current heavy users (>5 joints/week), 16 former regular users (no regular use for >3 months), and 59 never users. | General IQ, memory, processing speed, vocabulary, attention, and abstract reasoning. | Did not investigate cognitive functioning prior to cannabis use initiation. | After controlling for pre-drug performance, current heavy users performed worse than non-users on memory, processing speed, and general IQ. Current light users did not perform worse than non-users on any cognitive domain. | Did not investigate whether cannabis impairing effects were present at a later follow-up regardless of whether cannabis use continues, but did report that former regular users did not perform worse than non-users on any cognitive domain. | ANCOVA controlling for pre-drug performance with group as between-subject factor (current heavy, current light, former, and non-users) | SES, cigarette and alcohol use, and mental health diagnosis. |
| Hanson et al. 2010^33^ | 40 | 15-19 | 3 weeks | Cannabis measured at baseline. Cognition measured after 3 days, 2 weeks and 3 weeks of abstinence. | 2 groups: 19 users (>200 lifetime uses), 21 non-users (<5 lifetime uses). | Verbal memory, visual attention, verbal working memory, and time estimation. | Could not test if cognitive impairment was present prior to cannabis use because participants had already initiated cannabis when entering the study. | Did not test concurrent cognitive effects of cannabis. | No time by group interaction was observed for any of the cognitive tests. Across all domains tested, only attention accuracy remained deficient in users throughout the 3-week abstinence period. | Repeated-measures ANCOVA | Age and SES. |
| Infante et al. 2020^34^ | 175 | 12-15 | 14 y | Cannabis and cognition measured at each assessment (annual assessments). | Prevalence of use at baseline was 5%. | General IQ, processing speed, visuo-spatial abilities, memory, response inhibition. | Individuals more likely to use cannabis showed worse performance on response inhibition and visuo-spatial abilities. | No concurrent within-subject effects were significant for cannabis, such that increases in the number of days using cannabis at a given year were not associated with poorer cognitive functioning that same year. | Did not investigate whether cannabis impairing effects were present at a later follow-up regardless of whether cannabis use continues. | Hierarchical linear models disentangling between- from within-person differences. | Age, sex and recency of alcohol use. |
| Jacobus et al. 2015^35^ | 108 | 16-19 | 3 y (followed-up every 18 months) | Cannabis measured at baseline. Cognition measured at every assessment. | 2 groups: 49 cannabis users with concomitant alcohol use (>60 lifetime use) and 59 with limited substance use histories (<9 lifetime use). Over 80% of cannabis users continued to report >60 marijuana use episode per year by 3-year follow-up. | Complex attention/ working memory, verbal memory, processing speed, visuo-spatial abilities, and executive functioning. | Could not test if cognitive impairment was present prior to cannabis use because participants had already initiated cannabis when entering the study. | Group (users vs non-users) by time interactions were observed for learning/complex attention, and visuo-spatial functioning, such that cannabis users showed relative decline in performance compared to controls. Moreover, group (early onset vs late onset cannabis users - prior or after 16 years old) by time interaction was observed for learning/complex attention. Specifically, the early onset adolescents showed relative decline in performance compared to controls, while no differences were observed between late onset users and controls. | Cannabis users performed worse than controls across all three time points on various cognitive domains, and significant differences were more consistent at 19 years old (1.5-year follow-up) across domains, just prior to a narrowing substance use gap observed by age 20 (3-year follow-up) between users and controls for both alcohol and marijuana use. | Repeated-measures ANCOVA. | Lifetime alcohol use. |
| Lyketsos et al. 1999^36^ | 1318 | 18-64 | 12 y | Cannabis and cognition measured at wave 1 (baseline), wave 2 (1 year later), and at wave 3 (12 years later). | 3 groups based on their self-reported cannabis use at all 3 waves: 61% non-users (never used cannabis at all 3 waves), 18% persistent light users (never used daily), 10% persistent heavy users (reported daily use for over 2 weeks at least for one study wave). | Mini-Mental Status Exam | Could not test if cognitive impairment was present prior to cannabis use because participants had already initiated cannabis when entering the study. | Light and heavy cannabis users did not show greater decline on the mini-mental status exam than non-users. | Did not investigate whether cannabis impairing effects were present at a later follow-up regardless of whether cannabis use continues. | Linear regressions with MMSE change score (wave 3 - wave 2) as the dependent variable. | Sex, age, ethnicity, education. |
| McKetin et al. 2016^37^ | 1897 | 40-46 | 8 y (follow-up every 4 years) | Cannabis and cognition measured at all waves (baseline, 4 years later, and 8 years later). | 3 groups: no use, < weekly use, and > weekly use. | Verbal learning and memory, working memory, processing speed, and verbal intelligence. | Could not test if cognitive impairment was present prior to cannabis use because participants had already initiated cannabis when entering the study. | Between-person effect: using cannabis weekly or more often remained significantly associated with lower test scores on verbal learning. Within-person effects for cannabis use on verbal learning and memory were not significant, suggesting no significant difference in learning and memory during waves when cannabis using participants were using cannabis compared to waves when they were not using the drug. | Did not investigate whether cannabis impairing effects were present at a later follow-up regardless of whether cannabis use continues. | Random effects regression disentangling between- from within-subjects differences. | Baseline years of education, race, past alcohol consumption; as well as the following time-varying covariates: employment status, tobacco and alcohol use, exercise, depression and anxiety. |
| Meier et al.  2012^38^ | 874 | 7-13 | 25 y | Cannabis measured at ages 18, 21, 26, 32, 38. Cognition measured at baseline (age 13) and last follow-up (age 38). | 5 groups for persistence of cannabis dependence: never users, non-dependent users, those with 1 diagnosis of CUD, those with 2 diagnosis of CUD, those with 3 diagnosis of CUD. | General IQ, working memory, processing speed, perceptual reasoning and verbal comprehension. | No evidence that cognitive impairment was apparent prior to cannabis use initiation. | Persistent cannabis use from age 18-38 (at 3+ waves) was associated with IQ decline from age 7-13 to age 38. Greatest impairments found in executive function, memory, processing speed, and verbal comprehension. Adolescent-onset users showed greater IQ decline than adult-onset cannabis users: in fact, adult-onset cannabis users did not appear to experience IQ decline as a function of persistent cannabis use. | Among adolescent-onset persistent cannabis users, within-person IQ decline was apparent regardless of whether cannabis was used infrequently (median use = 14 days) or frequently (median use = 365 d) in the year before testing. This was not the case for adult-onset persistent cannabis users. | Ordinary least-squares linear regressions with cannabis use persistence as the independent variable and IQ change score as the dependent variable | Years of education. Redid main analysis when excluding people diagnosed with schizophrenia, those with persistent tobacco dependence, persistent hard-drug dependence, persistent alcohol dependence, past-week cannabis use, past 24-cannabis use. |
| Mokrysz et al. 2016^39^ | 2235 | 8 | 7 y | Cannabis use measured at age 15. Cognition: IQ measured at age 8 and 15. | 5 groups: 77% had never used, 11% had used < 5 times, 6% had used beteen 5-19 times, 3% had used between 20-49 times, 3% had used >50 times. | General IQ. | No evidence that cognitive impairment was apparent prior to cannabis use initiation. | Did not investigate whether cannabis impairing effects were present at a later follow-up regardless of whether cannabis use continues. | Cumulative cannabis use was negatively associated with IQ measured at the age of 15 when controlling for premorbid IQ, maternal factors, and mental health symptoms. However, this effect was attenuated when controlling for other substance use. | Linear least-squares regressions. | Cigarette and alcohol use, and childhood mental health symptoms. |
| Morin et al.  2019^40^ | 3659 | 13 | 4 y | Cognition and cannabis use measured every year for 4 years (13 to 16 y.o.). | Prevalence of cannabis use ranged from 4.6% at 13 y.o. to 28.8% at 16 y.o. | Non-verbal memory, response inhibition, working memory, non-verbal IQ (perceptual reasoning). | Individuals more likely to use cannabis showed lower response inhibition. | At the within-subject level, increases in cannabis use frequency in a given year were related to lower score on the non-verbal memory task in that same year. | At the within-subject level, increases in cannabis use frequency in a given year further predicted lower performance on the response inhibition task a year later. | Linear mixed effect models disentangling between-subject from within-subject differences. | SES, gender, ethnicity, and alcohol use. |
| Paige & Colder  2020^41^ | 387 | 11-12 | 9 y (annual follow-ups) | Cannabis measured at waves 1-4. Cognition measured at waves 1-3, and at waves 7-9. | Prevalence of use was 0% at wave 1, 1.6% at wave 2, 8.4% at wave 3 and 17.2% at wave 4. | Attentional and inhibitory control. | Overall, individuals more likely to use cannabis at an early age (12-14 years old) did not show worse levels of attentional and inhibitory control. | No concurrent effects were evaluated, only long-term. | High levels of early marijuana use at ages 12–14 significantly predicted low levels of adolescent attentional control at ages 18–21. | Prospective path analysis. | Alcohol use, antisocial problems. |
| Tait et al. 2011^42^ | 2404 | 20-24 | 8 y (follow-ups every 4 years) | Cannabis and cognition measured at each of the 3 waves. | 6 groups: 420 persistent never users, 657 always former users (had only used prior to baseline), 231 former light users (monthly at baseline, but former at 2nd follow-up), 60 former heavy users (weekly at baseline, but former at 2nd follow-up), 71 current light users (light users a baseline and 2nd follow-up), and 60 current heavy users (weekly use at baseline and 2nd follow-up). | Verbal learning and memory, verbal fluency, processing speed, and working memory. | Did not investigate cognitive functioning prior to cannabis use initiation. | A time (wave) by group interaction was found specifically for verbal memory. Never, always former and both the light and heavy former users had improved scores across all three waves, while the current light and current heavy users showed a decline in performance from wave 2 to wave 3. | A poorer development in performance from wave 1 (baseline) to wave 3 in current heavy users relative to former heavy users, suggesting that the impairing effects of cannabis are not long-term and resolve following abstinence. | Mixed-model repeated measures ANOVAs. | Education. |
| Co-twin designs | | | | | | | | | | | |
| Jackson et al. 2016^43^ | Study 1: 789; Study 2: 2277. | Study 1: 9-10; Study 2: 11-12. | Study 1: 10 y (5 follow-ups); Study 2: 7 y. | Cannabis measured at each assessment. Cognition measured at baseline and last follow-up. | Study 1: 60% users. Study 2: 36% users. | General IQ (verbal and non-verbal intelligence, visual processing). | Evidence for lower performance on verbal fluency tests, and to some extent visuo-spatial abilities, for those who later used cannabis. No evidence of cognitive vulnerability found for perceptual reasoning and visual processing. | When comparing siblings discordant for cannabis use, no differences in all IQ measures change between MZ and DZ siblings. | Did not investigate whether cannabis impairing effects were present at a later follow-up regardless of whether cannabis use continues. | Mixed-effects linear regression models. | Age, sex, race, zygosity, and SES. |
| Meier et al. 2018^44^ | 1989 | 12 y | 6 y | Cannabis measured at age 18. IQ measured at ages 5, 12, and 18. Executive functions measured at age 18. | At last follow-up (18 y.o.), 38% used cannabis in the past year, and 4% had a cannabis dependence. | General IQ, executive functions. | No evidence that cognitive impairment was apparent prior to cannabis use initiation among cannabis dependent twins and their non-dependent co-twin. | No evidence from discordant pairs that cannabis‐dependent adolescents had lower IQ or executive functions at any age. Among twin pairs, the more frequently cannabis‐using twin performed worse on 1 out of 6 executive function tests, but performed equally on IQ measures at any age then their co-twin. | Did not investigate whether cannabis impairing effects were present at a later follow-up regardless of whether cannabis use continues. | Linear regression with IQ change (from age 12-18) as dependent variable. | Sex, IQ at age 5 or 12. |
| Ross et al.  2020^45^ | 856 | 17 y | 6 y | Cannabis and cognition measured at baseline and follow-up. | At baseline, 7% with CUD. At follow-up 13% with CUD. | General IQ and executive functions modeled with bi-factor model (General EF factor and shifting-specific and updating-specific sub-factors). | Did not investigate cognitive functioning prior to cannabis use initiation. | No evidence for concurrent effects at the within-family level. | Among all tests measured and different ways of assessing cannabis use, there was evidence, at the within-family level, that an increase of cannabis use frequency at age 17 was associated with a decrease in general EF factor at age 23. | Multilevel models disentangling between-family difference and within-family difference. | Premorbid cognitive ability, sex, other substance use. |

**References**

1. D’Souza, D. C. *et al.* The psychotomimetic effects of intravenous delta-9-tetrahydrocannabinol in healthy individuals: Implications for psychosis. *Neuropsychopharmacology* **29**, 1558–1572 (2004).

2. Curran, V. H., Brignell, C., Fletcher, S., Middleton, P. & Henry, J. Cognitive and subjective dose-response effects of acute oral Δ9-tetrahydrocannabinol (THC) in infrequent cannabis users. *Psychopharmacology (Berl).* **164**, 61–70 (2002).

3. Hunault, C. C. *et al.* Cognitive and psychomotor effects in males after smoking a combination of tobacco and cannabis containing up to 69 mg delta-9-tetrahydrocannabinol (THC). *Psychopharmacology (Berl).* **204**, 85–94 (2009).

4. Weinstein, A. *et al.* A study investigating the acute dose-response effects of 13 mg and 17 mg Δ 9- tetrahydrocannabinol on cognitive-motor skills, subjective and autonomic measures in regular users of marijuana. *J. Psychopharmacol.* **22**, 441–451 (2008).

5. Hart, C. L., Van Gorp, W., Haney, M., Foltin, R. W. & Fischman, M. W. Effects of acute smoked marijuana on complex cognitive performance. *Neuropsychopharmacology* **25**, 757–765 (2001).

6. Ramaekers, J. G. *et al.* Cognition and motor control as a function of Δ9-THC concentration in serum and oral fluid: Limits of impairment. *Drug Alcohol Depend.* **85**, 114–122 (2006).

7. Spindle, T. R. *et al.* Acute Effects of Smoked and Vaporized Cannabis in Healthy Adults Who Infrequently Use Cannabis: A Crossover Trial. *JAMA Netw. open* **1**, e184841 (2018).

8. Adam, K. C. S., Doss, M. K., Pabon, E., Vogel, E. K. & de Wit, H. Δ9-Tetrahydrocannabinol (THC) impairs visual working memory performance: a randomized crossover trial. *Neuropsychopharmacology* (2020). doi:10.1038/s41386-020-0690-3

9. Heishman, S. J., Arasteh, K. & Stitzer, M. L. Comparative effects of alcohol and marijuana on mood, memory, and performance. *Pharmacol. Biochem. Behav.* **58**, 93–101 (1997).

10. Böcker, K. B. E. *et al.* Cannabis with high Δ9-THC contents affects perception and visual selective attention acutely: An event-related potential study. *Pharmacol. Biochem. Behav.* **96**, 67–74 (2010).

11. Hindocha, C. *et al.* Acute effects of delta-9-tetrahydrocannabinol, cannabidiol and their combination on facial emotion recognition: A randomised, double-blind, placebo-controlled study in cannabis users. *Eur. Neuropsychopharmacol.* **25**, 325–334 (2015).

12. Schoedel, K. A. *et al.* A randomized, double-blind, placebo-controlled, crossover study to evaluate the subjective abuse potential and cognitive effects of nabiximols oromucosal spray in subjects with a history of recreational cannabis use. *Hum. Psychopharmacol.* **26**, 224–236 (2011).

13. Morgan, C. J. A. *et al.* Individual and combined effects of acute delta-9-tetrahydrocannabinol and cannabidiol on psychotomimetic symptoms and memory function. *Transl. Psychiatry* **8**, (2018).

14. Arkell, T. R. *et al.* Cannabidiol (CBD) content in vaporized cannabis does not prevent tetrahydrocannabinol (THC)-induced impairment of driving and cognition. *Psychopharmacology (Berl).* **236**, 2713–2724 (2019).

15. Lawn, W. *et al.* Acute and chronic effects of cannabinoids on effort-related decision-making and reward learning: an evaluation of the cannabis ‘amotivational’ hypotheses. *Psychopharmacology (Berl).* **233**, 3537–3552 (2016).

16. Roser, P. *et al.* Psychomotor performance in relation to acute oral administration of Δ9-tetrahydrocannabinol and standardized cannabis extract in healthy human subjects. *Eur. Arch. Psychiatry Clin. Neurosci.* **259**, 284–292 (2009).

17. Morgan, C. J. A., Schafer, G., Freeman, T. P. & Curran, H. V. Impact of cannabidiol on the acute memory and psychotomimetic effects of smoked cannabis: Naturalistic study. *Br. J. Psychiatry* **197**, 285–290 (2010).

18. Englund, A. *et al.* Cannabidiol inhibits THC-elicited paranoid symptoms and hippocampal-dependent memory impairment. *J. Psychopharmacol.* **27**, 19–27 (2013).

19. Morgan, C. J. A. *et al.* Sub-chronic impact of cannabinoids in street cannabis on cognition, psychotic-like symptoms and psychological well-being. *Psychol. Med.* **42**, 391–400 (2012).

20. Colizzi, M. *et al.* Modulation of acute effects of delta-9-tetrahydrocannabinol on psychotomimetic effects, cognition and brain function by previous cannabis exposure. *Eur. Neuropsychopharmacol.* **28**, 850–862 (2018).

21. Ramaekers, J. G., Kauert, G., Theunissen, E. L., Toennes, S. W. & Moeller, M. R. Neurocognitive performance during acute THC intoxication in heavy and occasional cannabis users. *J. Psychopharmacol.* **23**, 266–277 (2009).

22. Theunissen, E. L. *et al.* Neurophysiological functioning of occasional and heavy cannabis users during THC intoxication. *Psychopharmacology (Berl).* **220**, 341–350 (2012).

23. D’Souza, D. C. *et al.* Effects of haloperidol on the behavioral, subjective, cognitive, motor, and neuroendocrine effects of Δ-9-tetrahydrocannabinol in humans. *Psychopharmacology (Berl).* **198**, 587–603 (2008).

24. D’Souza, D. C. *et al.* Blunted psychotomimetic and amnestic effects of Δ-9- tetrahydrocannabinol in frequent users of cannabis. *Neuropsychopharmacology* **33**, 2505–2516 (2008).

25. D’Souza, D. C. *et al.* Delta-9-tetrahydrocannabinol effects in schizophrenia: Implications for cognition, psychosis, and addiction. *Biol. Psychiatry* **57**, 594–608 (2005).

26. Henquet, C. *et al.* An experimental study of catechol-O-methyltransferase Val158Met moderation of Δ-9-tetrahydrocannabinol-induced effects on psychosis and cognition. *Neuropsychopharmacology* **31**, 2748–2757 (2006).

27. Auer, R. *et al.* Association between lifetime marijuana use and cognitive function in middle age the coronary artery risk development in young adults (CARDIA) study. *JAMA Intern. Med.* **176**, 352–361 (2016).

28. Becker, M. P. *et al.* Longitudinal changes in cognition in young adult cannabis users. *J. Clin. Exp. Neuropsychol.* **40**, 529–543 (2018).

29. Boccio, C. M. & Beaver, K. M. Examining the influence of adolescent marijuana use on adult intelligence: Further evidence in the causation versus spuriousness debate. *Drug Alcohol Depend.* **177**, 199–206 (2017).

30. Castellanos-Ryan, N. *et al.* Adolescent cannabis use, change in neurocognitive function, and high-school graduation: A longitudinal study from early adolescence to young adulthood. *Dev. Psychopathol.* **29**, 1253–1266 (2017).

31. Fried, P., Watkinson, B., James, D. & Gray, R. Current and former marijuana use: Preliminary findings of a longitudinal study of effects on IQ in young adults. *Can. Med. Assoc. J.* **166**, 887–891 (2002).

32. Fried, P. A., Watkinson, B. & Gray, R. Neurocognitive consequences of marihuana - A comparison with pre-drug performance. *Neurotoxicol. Teratol.* **27**, 231–239 (2005).

33. Hanson, K. L. *et al.* Longitudinal study of cognition among adolescent marijuana users over three weeks of abstinence. *Addict. Behav.* **35**, 970–976 (2010).

34. Infante, M. A. *et al.* Neuropsychological Trajectories Associated with Adolescent Alcohol and Cannabis Use: A Prospective 14-Year Study. *J. Int. Neuropsychol. Soc.* **26**, 480–491 (2020).

35. Jacobus, J. *et al.* Neuropsychological performance in adolescent marijuana users with co-occurring alcohol use: A three-year longitudinal study. *Neuropsychology* **29**, 829–843 (2015).

36. Lyketsos, C. G., Garrett, E., Liang, K. Y. & Anthony, J. C. Cannabis use and cognitive decline in persons under 65 years of age. *Am. J. Epidemiol.* **149**, 794–800 (1999).

37. McKetin, R., Parasu, P., Cherbuin, N., Eramudugolla, R. & Anstey, K. J. A longitudinal examination of the relationship between cannabis use and cognitive function in mid-life adults. *Drug Alcohol Depend.* **169**, 134–140 (2016).

38. Meier, M. H. *et al.* Persistent cannabis users show neuropsychological decline from childhood to midlife. *Proc. Natl. Acad. Sci. U. S. A.* **109**, (2012).

39. Mokrysz, C. *et al.* Are IQ and educational outcomes in teenagers related to their cannabis use? A prospective cohort study. *J. Psychopharmacol.* **30**, 159–168 (2016).

40. Morin, J. F. G. *et al.* A population-based analysis of the relationship between substance use and adolescent cognitive development. *Am. J. Psychiatry* **176**, 98–106 (2019).

41. Paige, K. J. & Colder, C. R. Long-Term Effects of Early Adolescent Marijuana Use on Attentional and Inhibitory Controlle. *J. Stud. Alcohol Drugs* **81**, 164–172 (2020).

42. Tait, R. J., Mackinnon, A. & Christensen, H. Cannabis use and cognitive function: 8-year trajectory in a young adult cohort. *Addiction* **106**, 2195–2203 (2011).

43. Jackson, N. J. *et al.* Impact of adolescent marijuana use on intelligence: Results from two longitudinal twin studies. *Proc. Natl. Acad. Sci. U. S. A.* **113**, E500–E508 (2016).

44. Meier, M. H. *et al.* Associations between adolescent cannabis use and neuropsychological decline: a longitudinal co-twin control study. *Addiction* **113**, 257–265 (2018).

45. Ross, J. M. *et al.* Investigating the causal effect of cannabis use on cognitive function with a quasi-experimental co-twin design. *Drug Alcohol Depend.* **206**, (2020).
